# Chronic Electrical Stimulation Promotes the Excitability and Plasticity of ESC-derived Neurons following Glutamate-induced Inhibition *In vitro*

**DOI:** 10.1038/s41598-018-29069-3

**Published:** 2018-07-19

**Authors:** Charles-Francois V. Latchoumane, LaDonya Jackson, Mohammad S. Eslampanah Sendi, Kayvan F. Tehrani, Luke J. Mortensen, Steven L. Stice, Maysam Ghovanloo, Lohitash Karumbaiah

**Affiliations:** 1 Regenerative Bioscience Center, ADS Complex, University of Georgia, Athens, Georgia; 2 School of Chemical, Materials, and Biomedical Engineering, College of Engineering, University of Georgia, Athens, Georgia; 3 School of Electrical and Computer Engineering, Georgia Institute of Technology, Atlanta, Georgia

## Abstract

Functional electrical stimulation (FES) is rapidly gaining traction as a therapeutic tool for mediating the repair and recovery of the injured central nervous system (CNS). However, the underlying mechanisms and impact of these stimulation paradigms at a molecular, cellular and network level remain largely unknown. In this study, we used embryonic stem cell (ESC)-derived neuron and glial co-cultures to investigate network maturation following acute administration of L-glutamate, which is a known mediator of excitotoxicity following CNS injury. We then modulated network maturation using chronic low frequency stimulation (LFS) and direct current stimulation (DCS) protocols. We demonstrated that L-glutamate impaired the rate of maturation of ESC-derived neurons and glia immediately and over a week following acute treatment. The administration of chronic LFS and DCS protocols individually following L-glutamate infusion significantly promoted the excitability of neurons as well as network synchrony, while the combination of LFS/DCS did not. qRT-PCR analysis revealed that LFS and DCS alone significantly up-regulated the expression of excitability and plasticity-related transcripts encoding N-methyl-*D*-aspartate (NMDA) receptor subunit (NR2A), brain-derived neurotrophic factor (BDNF) and Ras-related protein (RAB3A). In contrast, the simultaneous administration of LFS/DCS down-regulated *BDNF* and *RAB3A* expression. Our results demonstrate that LFS and DCS stimulation can modulate network maturation excitability and synchrony following the acute administration of an inhibitory dose of L-glutamate, and upregulate *NR2A, BDNF* and *RAB3A* gene expression. Our study also provides a novel framework for investigating the effects of electrical stimulation on neuronal responses and network formation and repair after traumatic brain injury.

## Introduction

Direct current stimulation (DCS) has gained increasing attention in clinical and cognitive neuroscience applications due to it being a relatively inexpensive, non-invasive and easily parametrizable tool with minimal adverse effects^1,2^. Transcranial DCS (tDCS) has been demonstrated to mediate therapeutic outcomes^1,3,4^ in depression^5–7^, dementia^8,9^, pain^10,11^ and central nervous system injury^12–14^. In addition, tDCS applications extend to neuroprosthetics including improving working memory^15^, motor and declarative learning^16–19^ and decision making and addiction^20–23^. In the healthy brain, the effects of tDCS and low frequency stimulation (LFS) on neuronal function and plasticity are thought to be mediated by changes in excitation/inhibition balance^24–26^, modulation of the TrekB/BDNF pathway^16^, NMDA receptor interactions and intracellular calcium concentration^27–29^. However, the underlying molecular, cellular and network mechanisms of action of tDCS remain largely unknown.

Traumatic brain injury (TBI) is a complex neuropathology commonly affecting healthy youth and war veterans^30,31^, with a wide range of severity and phenotypic outcomes^32^. The progression of TBI pathophysiology begins with the primary insult, which results in the acute impact induced mechanical damage to brain tissue. The secondary phase that follows involves inflammation, oxidative stress, metabolic dysregulation and excitotoxic release of neurotransmitters such as glutamate^32–34^; resulting in significant brain tissue loss and functional impairments. To this date, no therapeutic approaches have proven to be effective in treating TBI^32^. tDCS has demonstrated significant promise in mediating functional recovery after TBI in preclinical studies^35^, and in treating and rehabilitating brain function in TBI patients^12,36,37^. The growing usage of tDCS as a therapeutic tool for TBI calls for a fundamental understanding of the underlying mechanisms of this new approach that would favor the development of more optimized protocols with temporally targeted applications^38–40^.

Microelectrode array (MEA) recordings of neuronal cell cultures have been suggested as a promising proxy to the study of brain neural networks^41^. MEA studies have helped characterize neuronal network maturation^42^ and disruption under various pharmacological treatments^43–45^, and could provide for realistic models to evaluate neuronal tissue development, disorders and injuries^41^. The combination of DCS and MEA recordings to study the response of impaired neural networks remains underexplored, and could potentially help reveal important features and underlying mechanisms of action of electrical stimulation on brain tissue^46^.

Here, we used embryonic stem cell (ESC)-derived neuron and glia co-cultures in combination with current stimulation protocols (i.e. DCS and LFS) and MEA recordings to investigate and modulate the response of neuronal networks to L-glutamate induced inhibition. Neurons and glia are both essential for the development and maintenance of synaptic connectivity within neural networks^47,48^. Stem cells have begun to gain popularity as cells of choice to study neural network activity due to their ability to differentiate into both neurons and glia, and owing to their uniform clonal properties that can help minimize experimental variability^49–51^. ESC-derived co-cultures containing astrocytes, oligodendrocytes, and a variety of neuron types can therefore serve as a model system to better understand baseline neural network function, and to investigate neuromodulatory mechanisms of recovering network activity after toxic exposure to excitatory neurotransmitters *in vitro*^52–54^.

In this study, we present a model to study the impact of electric stimulation on the formation of ESC-derived neural networks following glutamate-induced inhibition. We quantified the impact of glutamate treatment on the rate of maturation in excitability and synchrony of neural networks as well as following three protocols based on chronic DCS: 1) DCS (one time cathodal and daily anodal DCS; 6 days); 2) LFS low frequency stimulation (daily 0.1 Hz DCS stimulation; 5 days); and 3) a combination of DCS and LFS. We quantified the effects of stimulation on changes in network responses, and the change in excitability and synaptic function-related expression of *NR2A*, *NR2B*, *BDNF*, and *RAB3A* transcripts using qRT-PCR.

## Materials and Methods

### Cell culture

Mouse Hb9 ESC-derived neuron and glial cells (ArunA Biomedical, GA; Cat 7025) were cultured in AB2 basal neural medium (ArunA Biomedical, GA) and Dulbecco’s Modified Eagles Medium/F12 (DMEM/F12; Corning, NY) following a previously published protocol^55^. Briefly, each well of 12 well/64 electrode per well containing MEA plates (Axion Biosystems, GA), or glass bottom petri dishes (Cellvis, CA) were coated with polyethylenimine (PEI) (Sigma-Aldrich, MO) and incubated for 1 h at 37 °C and 5% CO_2_, then washed with deionized water and allowed to air dry overnight. The surfaces were subsequently coated with 20 µg/ml laminin (ThermoFisher Scientific, MA) for 2 h at 37 °C and 5% CO_2_ immediately prior to seeding. The cells were subsequently seeded in triplicate on either glass-bottom petri dishes, or in 12 well MEA plates. MEA plates (12-well, 1.43 × 1.43 mm surface) were seeded at a density of 40,000 cells per well, and glass-bottom petri dishes were seeded at a density of ~125,000 per well to maintain comparable cell seeding density to MEAs.

### L-glutamate Inhibition Assays

Two weeks post-seeding, neuronal populations in glass-bottom petri dishes and MEA plates were treated with media only (controls) or with media containing 100 µM L-glutamate (Fisher Scientific, NH) and incubated at 37 °C for a period of 20 min. Following this brief exposure, medium was removed and replaced with fresh complete growth medium devoid of excitotoxic agent and returned to the incubator. These cells were cultured for an additional week during which time electrophysiological assessments were made as described below. The cells were processed for immunocytochemical, and qRT-PCR analyses as described below at the experimental endpoint.

### Electrophysiology Experiments and Analysis of Neural Metrics

Cellular electrophysiological activity was recorded for 5 min each at day 7, 14, and at day 21 from control and L-glutamate treated groups subjected to no stimulation, LFS-only, DCS- only, and LFS/DCS treatments. Recordings were acquired using the Maestro system (Axion Biosystems, GA) set at 37 °C, and analyzed using Axis software (Axion Biosystems, GA). After data acquisition and recording, the media was removed and the wells were replenished with fresh media before returning the plate to the incubator.

Data obtained from each individual channel (electrode) was sampled with a gain setting of 1200× and a sampling rate of 12.5 kHz. The raw recorded local field potentials (LFP) were filtered using a Butterworth bandpass filter (200–3000 Hz cutoff frequencies) for all analyses. Event-triggered potentials were detected from the bandpass filtered data using a 6× standard deviation root mean square (RMS) threshold on each channel and all triggered waveforms were saved on file along with raw LFP data. An optional spike sorting was performed for electrode showing multi-unit activity using principal component analysis (PCA) decomposition of waveform and k-nearest neighbor (KNN) clustering using custom MATLAB scripts (Mathworks, Natick, MA). Offline analyses were performed using custom MATLAB scripts for spike properties (peak-to-valley amplitude and width, mean firing rate, mean bursting rate) and network properties (# of units per well, # of active electrode per well, # of bursting cells per well, mean population firing rate, mean population bursting rate, event synchronization and spike train cross-correlation peak).

### Measures of excitability

For each recording electrode (64 channels per well), we estimated the number of triggered spikes and classified the electrode as active if at least 5 spikes/min were observed over the 5 minutes of recording^44^. The total number of active electrodes was then derived for each well.

The weighted mean firing rate (wMFR) can estimate the overall population excitability and connectivity, and was derived as the mean firing rate of a well, averaged over the active electrodes (wMFR is expressed as spikes per sec per active electrode). The instantaneous wMFR is derived after binning the activity of all electrodes (bin size: 100 msec) and is estimated as spike per bin and normalized to the number of active electrodes.

For each spiking unit/electrode, bursting was estimated following low-threshold rebound burst criteria: 50 msec of no spiking (silencing period), followed by at least 3 spikes with an inter-spike interval lower than 5 msec. The maximum inter-spike interval was set to 20 msec within one burst. The number of bursting cells per well was estimated from the number of spiking units/electrodes showing low-threshold burst.

### Measures of Synchrony

Population burst estimation was based on the instantaneous wMFR and the instantaneous count of active electrode per time bin (CellCount per Bin)^56–58^. A population burst was counted if: 1) at least 30% of active electrodes were determined to be spiking together within a 100 msec bin, and 2) the wMFR for that same bin was higher than 0.5 spikes/bin/electrodes. The start and stop of the burst were estimated from a threshold crossing. The threshold value for burst start and stop was estimated from the cumulative histogram of the product of instantaneous wMFR and CellCountBin (50%). The mean population burst firing rate (mBFR) was then estimated as the count of burst event over the entire recording duration (burst event/sec).

Event synchronization^59,60^ was obtained from the average event synchronization value over all unique pairs of spiking units/electrodes (only active electrodes were considered for the estimation). Briefly, for a single pair of electrodes, the event synchronization was derived from the number of spiking events that appeared within a short window of time (quasi-simultaneous) and normalized to be: a) symmetrical (i.e. no directionality), and b) unitary for identical spike trains (i.e. spikes showing identical and asynchronous spike train would have an event synchronization value of 1 and 0, respectively).

The cross-correlation based synchrony (cross-correlation peak) is derived from the average cross-correlation peak over all unique pairs of active electrodes^61,62^. For each unique pair, the cross-correlogram histogram (range [−10, 10] sec; bin size = 100 msec) is estimated and normalized (normalization based on the square root of the product of the two spike trains length). The cross-correlation peak is the value of the normalized cross-correlogram at lag = 0.

### Low Frequency and Direct Current Stimulation

LFS was administered through the electrodes in each well using the Maestro system. The Axis software was programmed to deliver monophasic anodal LFS at 10 µA and at a frequency of 0.1 Hz for 15 minutes every day from days 15–19^16^. Monophasic cathodal and anodal DCS was administered from a battery powered external device that contained variable resistors set to deliver 10 µA through a Keithley Series 2280 power supply (Keithley Instruments, OH) and a floating ground. Cathodal DCS was administered at the onset of excitotoxicity on Day 14 (15 min), and anodal DCS was administered from day 15–19 for 15 min/day. The initial cathodal DCS stimulation has been suggested to reduce the extent of glutamate-induced hyper-excitability and cytotoxicity, and its impact on brain recovery following injury^63,64^. Day 15 to 19 stimulations (LFS and DCS anodal) were designed to entrain the neural network into an excitable state, which is hypothesized to induce its maturation^65,66^. For clarity, we assigned the following labels to the different groups: FES protocol without L-glutamate were labelled NoStim (control), LFS, DCS and LFS/DCS in control condition. FES following L-glutamate infusion were labelled NoStim L-glutamate, LFS, DCS and LFS/DCS L-glutamate conditions.

### Immunocytochemistry

Cells seeded in glass bottom petri-dishes were fixed in 4% paraformaldehyde containing 0.4 M sucrose in phosphate buffered saline (PBS) for 20 minutes at 1 and 3 weeks post-seeding. They were then washed thrice in PBS and permeabilized in blocking buffer (PBS containing 4% goat serum, 0.5% Triton-X100) for 1 h. Cells were then incubated with blocking buffer containing primary antibodies overnight at 4 °C. Primary antibodies specific to β-III tubulin (1:200; Millipore, MA), Sox-1 (1:500; R&D Systems, MN), GFAP (1:500; Dako Agilent, CA) and O4 (1:500; R&D systems, MN) were used to mark neurons, neural stem cells, astrocytes, and oligodendrocytes, respectively. The next day, the cells were rinsed thrice with PBS, and incubated with blocking buffer for 1 h. Appropriately matched 555 anti-Mouse IgM (1:220; ThermoFisher Scientific, MA), 647 Goat anti-Mouse IgG1 (1:220; ThermoFisher Scientific, MA), 555 Goat anti-Rabbit IgG (1:220; ThermoFisher Scientific, MA), and 488 anti-Chicken IgY (1:220; ThermoFisher Scientific, MA) secondary antibodies in blocking buffer were then applied for 1 h. The cells were then again washed thrice with PBS then incubated in a nuclear stain (NucBlue; ThermoFisher Scientific, MA) for 5 min, and washed thrice with PBS. The cells were mounted with fluoromount-G (SouthernBiotech, AL), coverslipped, and imaged using a Leica DMIRBE fluorescence microscope (Leica Microsystems Inc, IL).

### qRT-PCR Analysis

Total RNA was isolated using RNeasy Plus Mini kit (Qiagen, CA) on day-21 from LFS or DCS stimulated and unstimulated cells belonging to control (no glutamate exposure) and treated (exposed to 100 µM L-glutamate), respectively (Table 1). Total RNA was quantified using a NanoDrop 8000 (ThermoFisher Scientific, MA), and cDNA was synthesized using the RT First Strand cDNA synthesis kit (Qiagen, CA). A total of 100 ng total RNA equivalent of cDNA template was used in 25 µL qRT-PCR reactions for each treatment group along with SYBR green dye (Qiagen, CA), and pre-validated primers targeting mouse *NR2A, NR2B, BDNF, RAB3A*; and the endogenous housekeeping genes *GAPDH* and *HPRT1* (Qiagen, CA) and amplified using a ABI 7900HT qRT-PCR instrument (Applied Biosystems, CA) using conditions described previously^67,68^. Each sample was assayed in triplicate using cycle conditions: 95 °C for 10 minutes, 40 cycles of 95 °C for 15 seconds, and 60 °C for 1 minute, followed by a melting curve analysis. Relative quantitative gene expression was determined using the ΔΔCT method. The fold increase or decrease in target gene expression was calculated after normalization to media–only control and against endogenous controls for each sample and then presented as relative units.

**Table 1:** Pre-validated mouse qRT-PCR primers used to measure gene expression at week 3.

Gene	Symbol	Refseq Number
*NR2A*	Grin2a	NM_008170
*NR2B*	Grin2ab	NM_008171
*BDNF*	BDNF	NM_001048139
*RAB3A*	RAB3A	NM_001166399
*GAPDH*	GAPDH	NM_008084
*HPRT1*	HPRT	NM_013556

### Statistical Analysis

Analyses of immunocytochemical staining and cell quantification was performed using Volocity software (PerkinElmer, MA). Unless stated otherwise all results are expressed as mean +/−SEM. Repeated measure ANOVA, two-way and one-way ANOVA were performed for longitudinal and group difference test with post-hoc correction for multiple comparisons when appropriate (Holm-Sidak). When necessary (normality test fail or equality of variance test fail), nonparametric alternative test were performed, i.e. Kruskal-wallis test (with post-hoc Dunn-Sidak correction), Wilcoxson ranksum test and Signrank test. Distribution comparisons were performed either using the Kolmogorov-Smirnov test (Lillie’s correction), Shapiro-Wilk test, or the Chi-square test goodness-of-fit test. Differences were considered statistically significant if p < 0.05. All statistical tests were performed using either SigmaPlot (Systat Software inc., CA) or MATLAB^®^ statistical tool box (MathWorks Inc., MA).

### Data availability

All datasets generated or analyzed in the current study are available from the corresponding author on reasonable request.

## Results

### ESC-derived neurons and glia mimic the cellular composition of mature neural tissue

In this study, we used mouse ESC-derived neurons and glia in order to investigate the maturation and composition of neural tissue (See Materials and Methods section for details).

**Figure 1:**
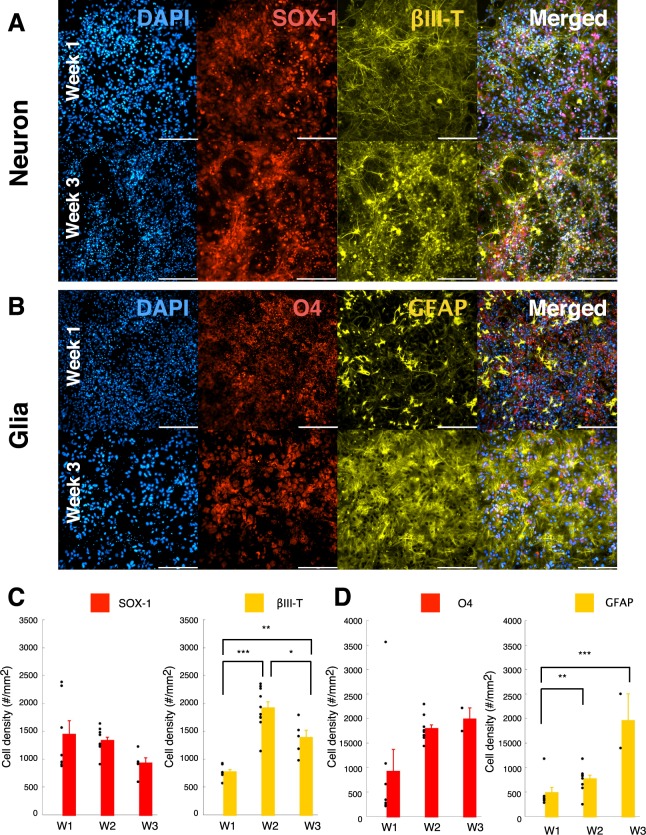
ESC-derived neurons and glia mimic the cellular composition of mature neural tissue. (**A**) ESC-derived neuronal population for the control condition (CTR, no treatment) at week 1 (top panels) and week 3 (bottom panels). Panels from left to right are DAPI, SOX-1 (Neural progenitor origin marker), βIII-Tubulin (Neuronal marker) and merged image representing all 3 fluorescent markers. Scale = 200 µm. (**B**) ESC-derived glial population for the control condition (CTR, no treatment) at week 1 (top panels) and week 3(bottom panels). Panels from left to right indicate DAPI, O4 (Oligodendrocyte marker), GFAP (Glial marker) and merged image representing all 3 fluorescent markers. Scale = 200 µm. (**C**) Neuronal cell density (#/mm2) estimated for the control condition (CTR) over week1 through week3. Quantification of the SOX-1 marker (Neural Stem Cell marker; left panel) and Beta-III tubulin (Neuronal marker; right panel). (**D**) Glial cell density (#/mm2) estimated for the control condition (CTR) over week1 through week3. Quantification of GFP marker (Differentiated motor neurons; left panel), O4 marker (Oligodendrocyte marker; middle panel) and GFAP (Astrocyte marker; right panel). Data is shown as scatter plot of individual plate quantifications and bar plot representing mean and s.e.m. * indicates p < 0.05, ** indicates p < 0.01 and *** indicates p < 0.001 using post-hoc multiple comparison with Holm-Sidak correction.

We found a characteristic differentiation (Fig. 1A) of the ESC-derived cells as early as week 1, into β-III tub+ (neuronal specific marker) neurons and SOX-1+ (a neuronal marker for cell with progenitor stem cell origin) cells. The ESC-derived cells also showed positive differentiation into oligodendrocytes (Fig. 1B, O4+) and astrocytes (Fig. 1B, GFAP+). These observations were confirmed by cell density quantification (Fig. 1C, D). We found that over three weeks of maturation, ESC-derived neurons demonstrated an increased differentiation toward βIII-tub+ neurons that plateaued at week 2 (Fig. 1C), whereas ESC-derived glia a steadily differentiated into O4+ and GFAP+ cells up until week3 (Fig. 1D).

The results indicate that the ESC-derived neurons and glia mature to a dynamically increasing density of βIII-tub+ neurons, O4+ oligodendrocytes, and GFAP+ astrocytes over a period of three weeks *in vitro*.

### ESC-derived neurons and glia form interconnected neural networks

**Figure 2:**
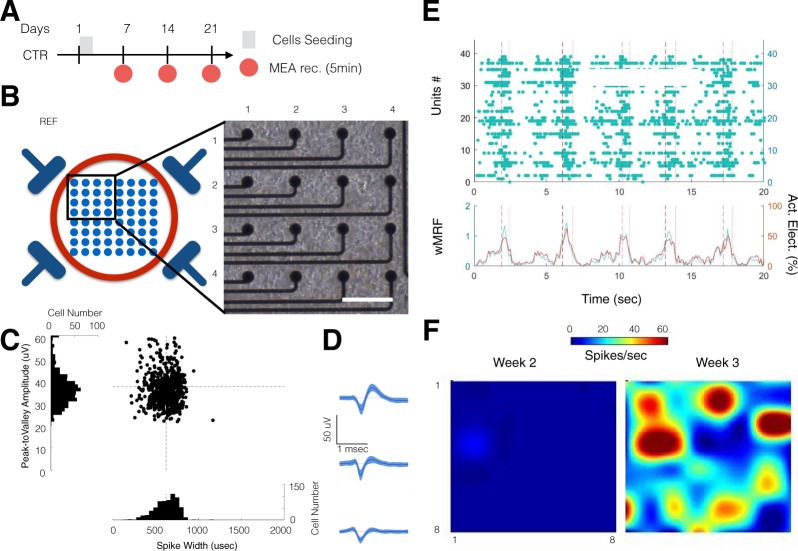
ESC-derived neurons and glia form functional neural network on MEA. (**A**) Experimental schedule for neural stem cell seeding and culture. For the control group, recordings were performed from week1 to week 3. (**B**) Representative micro-electrode array (MEA) recording setup. Schematic of the bottom of the well with 4 reference electrodes and 8 × 8 electrode array (left). Top left quadrant of a 64-channel MEA plate, 2 weeks after stem cells seeding (right; Scale = 100 μm). (**C**) Scatter distribution of the spike width (µsec; x-axis) against the peak-to-valley amplitude (µVolt; y-axis) for individual unit recorded from control condition at week 3. The distribution (cell count per bin) is displayed as a projection of each axis. The dashed black line represents the average of spike width (vertical) and peak-to-valley amplitude (horizontal). (**D**) Representative average spike wave forms obtained from 3 electrodes at week 3 from the control group. Data is shown as mean and s.e.m. (**E**) Raster plot of one well recorded at week 3 (top panel). The population instantaneous firing rate (left y-axis in green; wMFR, spikes/bin/electrode) and the percentage of active electrodes per bin (right y-axis, in red; active electrode in %). The vertical dashed and dotted lines indicate the start and stop of a detected population burst, respectively. (**F**) Heat map showing the evolution of the average activity in a control well from week 2 to week 3.

In order to characterize the integrity and functionality of the neural network formed using the ESC-derived neurons and glia, we used MEA recording assays (Fig. 2B). ESC-derived cells were seeded at day 1 in each well of 12-well MEA plates, and recordings were acquired at weeks 1 (7 days following seeding), 2 and 3 (Fig. 2A).

We observed that the distribution of spike width (peak-to-valley width: 553 ± 150 µsec) and spike amplitude (peak-to-valley amplitude: 37 ± 12 µVolt) was normal (Fig. 2C) and typical of neuronal spike waveforms (Fig. 2D)^69,70^.

We quantified the functional connection between active electrodes/units using event synchronization^59,60^ and cross-correlation peak measures^61,62^. As expected, ESC-derived neurons and glia formed networks that exhibited synchronous activity (Fig. 2E), and with a high tendency for network bursting (See materials and methods for details).

**Figure 3:**
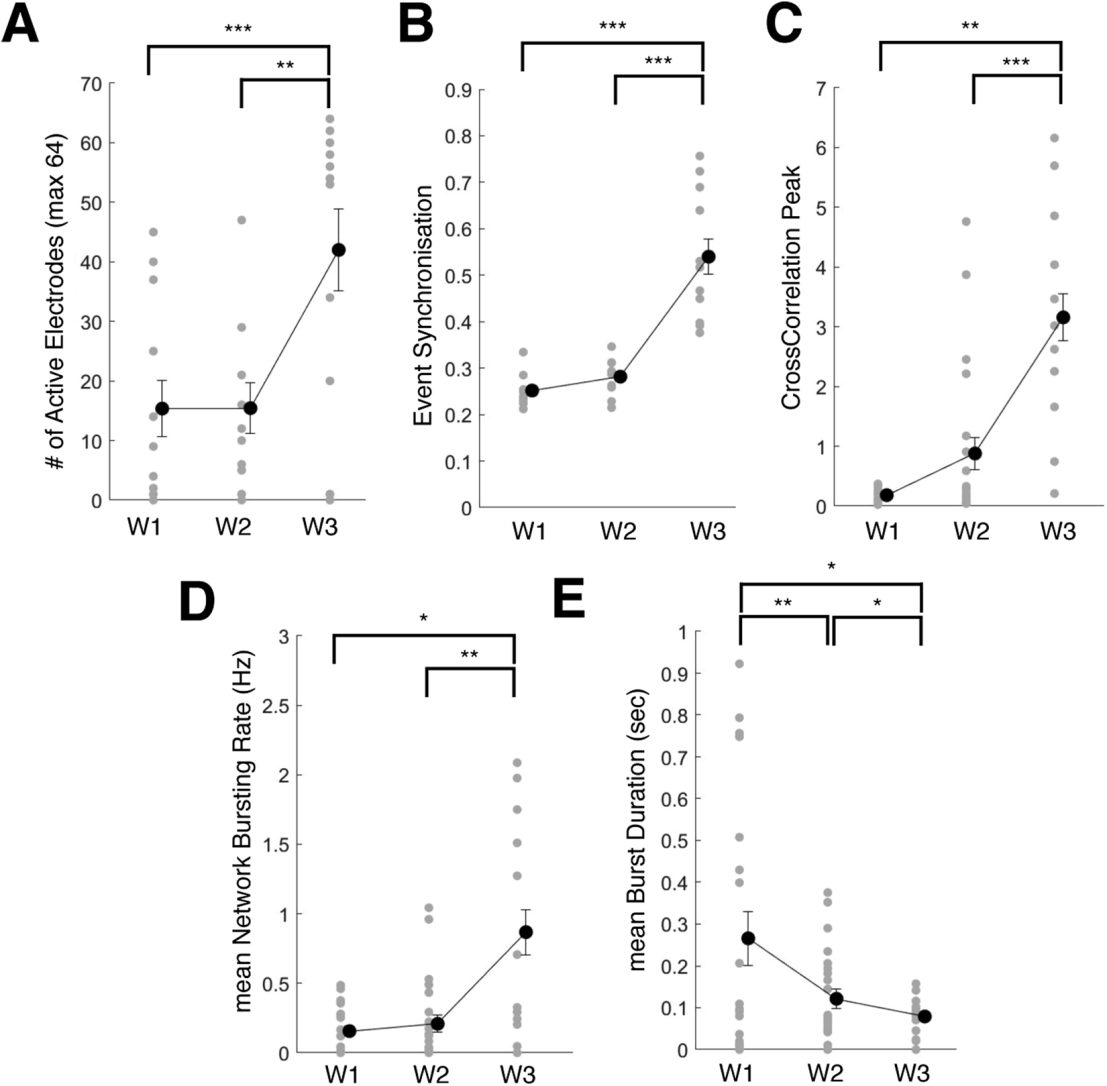
Temporal maturation of ESC network connectivity (**A**) Change in number of active electrodes quantified from week 1 through week 3 in control condition. (**B**) Change in Event Synchronization values from week 1 through week 3 in control condition. (**C**) Change in Cross-correlation Peak values from week 1 through week 3 in control condition. (**D**) Change in mean Network Bursting Rate values from week 1 through week 3 in control condition. (**E**) Change in mean Burst Duration values from week 1 through week 3 in control condition. Data is shown as scatter plots of individual well quantifications and error bars representing mean and s.e.m. * indicates p < 0.05, ** indicates p < 0.01 and *** indicates p < 0.001 using post-hoc multiple comparison with Holm-Sidak correction.

Consistently with the observed neuronal differentiation, the activity observed at each electrode increased over time (Supplementary Fig. 1). The number of active electrodes (i.e. electrode with spiking rate >5 spikes/min) per MEA (1 well, 64 channels) showed a significant increase from week 1 through week 3 (Fig. 3; repeated-measure ANOVA, time: p < 0.001; post-hoc Holm-Sidak correction: pw2-w3 < 0.01 and pw1-w3 < 0.001). ESC-derived neural networks showed increasing synchrony over time (Fig. 3B,C; repeated measure ANOVA, time: p < 0.001 for both event synchronization and cross-correlation peak). In addition, we observed that over time, the network bursting rate showed a marginal increase (repeated measure ANOVA, time: p = 0.07) and the mean burst duration significantly decreased over time (repeated measure ANOVA, time: p < 0.001).

Altogether, these results indicate that the ESC-derived neuronal and glial cells mature into a connected neural network that demonstrate a temporal increase in synchronization and population bursting.

### L-glutamate treatment impaired network maturation excitability and synchrony

**Figure 4:**
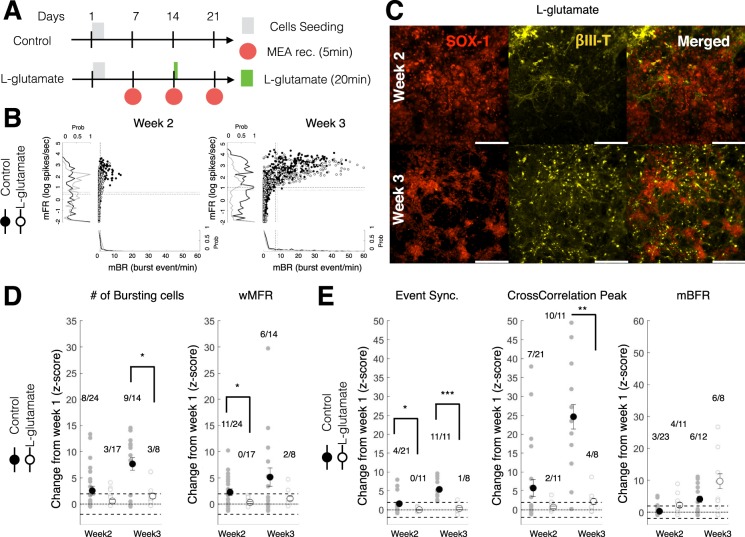
Acute L-glutamate treatment impaired network activity and synchrony immediately and over a week post-administration. (**A**) Experimental schedule for ESC-derived neuron seeding, culture and L-glutamate treatment. In the treatment group, 100 µM of L-glutamate was administered at day14 for 20 min then washed out. MEA recordings were performed from week 1 to week 3. (**B**) Scatter distribution of the mean burst firing rate (mBR; Burst event/min; x-axis) against the mean firing rate (mFR; log of spike/sec; y-axis) for individual unit recorded from CTR (black filled circles) and L-glut (black open circle) groups. Data is shown for week 2 (left panel) and week 3 (right panel). The normalized distribution CTR (black line) and L-glut (gray line) groups are displayed as a projection of each axis. The dashed black line represents the average for the CTR group. The dotted black line represents the average for the L-glut group. mFR: mean firing rate for individual electrodes; mBR: mean bursting rate for individual electrodes. (**C**) ESC-derived neurons after L-glutamate treatment (L-glut; 100 µM for 20 min at day 14) at week 2 (top panels) and week 3 (bottom panels). From left to right panels are shown SOX-1, βIII-Tubulin and merged image of both fluorescent markers. Scale = 200 μm. (**D**) Change at week 2 and week 3 (expressed as a Z-score from week1 baseline) of the number of bursting cells (left panel) and weighted mean firing rate (wMFR; right panel). For each group, the number of wells with significantly increasing change over the total number of wells recorded is shown above the scatter plot. wMFR: weighted mean population firing rate. (**E**) Change in network synchrony at week 2 and week 3 (expressed as a Z-score from week1 baseline) for event synchronization (left panel), cross-correlation peak (middle panel) and mean network burst firing rate (mBFR; right panel). For each group, the number of wells with significantly increasing change over the total number of wells recorded is shown above the scatter plot. For post-hoc two-sample test *, ** and *** indicate p < 0.05, p < 0.01 and p < 0.001, respectively. mBFR: mean population burst firing rate.

We then investigated the effect of L-glutamate exposure on the maturation and connectivity of neural networks derived from ESC-derived neurons and glia. L-glutamate treatment was administered 2 weeks post-seeding (Fig. 4A; day14: 20 min exposure then wash; 100 µM). This dose was chosen to affect network maturation and plasticity without inducing complete silencing or critical impairment of network formation^71^.

The immunohistochemical characterization of neuron/glial co-cultures following exposure to L-glutamate did not show any qualitative differences in comparison to control untreated cells. Both week 2 and week 3 immunohistochemical staining following L-glutamate exposure showed the presence of βIII-tub+ and SOX-1+ cells (Fig. 4C). Similarly, O4+ and GFAP+ glial cells were also prominently evident within the co-cultures following L-glutamate treatment (Supplementary Fig. 2B).

The individual characterization of each unit/electrode revealed a progressive increase in the firing rate (mFR: mean firing rate) and bursting rate (mBR: mean bursting rate) from week 2 to week 3 (Fig. 4B). We quantified the rate of change from week 1 (baseline prior to L-glutamate treatment) for measures of excitability (# of bursting cells and wMFR) as well as measures of synchrony (Event synchronization and Cross-correlation Peak) by computing the z-scores at week 2 and week 3. We found that L-glutamate treated wells showed a significantly lower z-score for measures of excitability than the control wells (repeated-measure ANOVA, group: p < 0.05) particularly at week 2 (wMFR, p < 0.05, Student t-test) and at week 3 (# of cell bursting, p < 0.05, Student t-test). The control wells had a proportion of wells that demonstrated a significant increase in excitability from week 1 (Z-score >1.96 or p < 0.05) above 33% (range 33.3 to 64.3%) when compared to L-glutamate treated cells, which demonstrated lower excitability (<38%; range 0 to 37.5%) as indicated by the number of bursting cells and the wMFR (Fig. 4D; well ratio).

Similarly, we found that L-glutamate treated wells showed a significantly reduced rate of change in synchrony measures (repeated-measure ANOVA, group: p < 0.05) particularly at week 2 (Event Synchronization, p < 0.05; Student t-test) and at week 3 (Event Synchronization, p < 0.05; Cross-correlation peak: p < 0.01; Student t-test) when compared to control untreated cells. No significant differences were observed in the rate of network bursting (mBFR; repeated-measure ANOVA, group: p = 0.927). At week3, we observed that L-glutamate treated cells demonstrated significantly lower measures of synchrony (Event Synchrony: 1/8 or 12.5%; Cross-correlation Peak: 4/8 or 50%) when compared to the untreated controls (Event Synchrony: 11/11 or 100%; Cross-correlation Peak: 10/11 or 90.1%).

Importantly, no differences were observed in the rate of change in the number of active electrodes between control and L-glutamate treated wells (repeated-measure ANOVA, group × time: p = 0.298).

Altogether these results indicate that L-glutamate treated wells showed a reduced maturation in excitability and network connectivity immediately (immediately following L-glutamate treatment) and over a week following exposure, without affecting the rate of change in the number of active electrodes.

### Electrical stimulation helps sustain network activity

Since DCS and LFS approaches have been demonstrated to induce functional recovery following CNS injury^12,72–74^, we investigated the effects of electrical stimulation on the rate of change in the network excitability and connectivity after network inhibition by L-glutamate (Fig. 4A,B).

**Figure 5:**
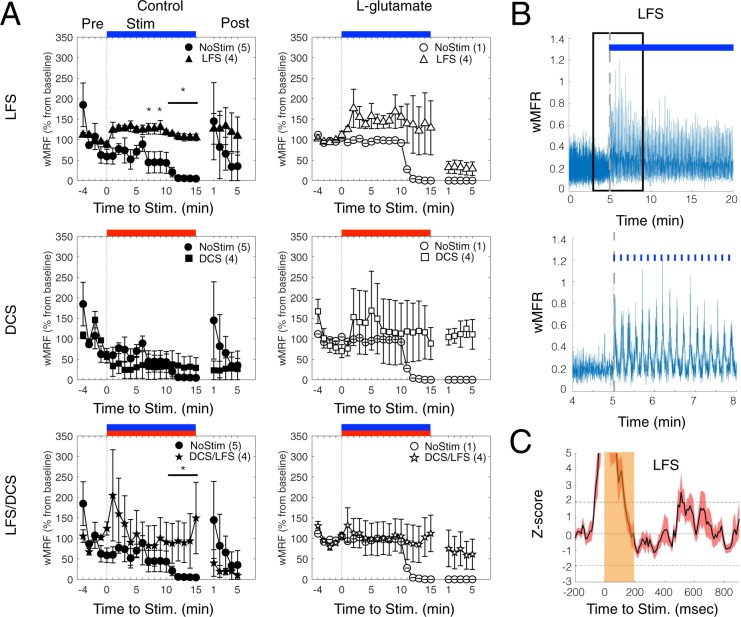
Evoked Network Response from different FES protocols. (**A**) Average wMFR as a percentage of the baseline (Pre) during the pre-stimulation, stimulation (Stim, marked by blue, red and blue/red colored bars for LFS, DCS and LFS/DCS, respectively) and post-stimulation (Post) period. The dashed dark lines indicates the start of the 15 min electrical stimulation. * indicate p < 0.05, ranksum between NoStim and Stimulation. (**B**) Representative trace showing the instantaneous wMFR (100 msec binning, stimulation duration 15 min) for the LFS condition (upper panel; pulse of 200 msec duration at 0.1 Hz, blue marks/bar) and a magnified representation of a 4 min section of activity (lower panel; marked with a black box in the upper panel); dashed gray line indicate the start of stimulation. (**C**) Average peri-stimulus histogram of the population activity (pulse duration: 200 msec) expressed as a z-score (baseline: 200 msec prior stimulation; Average of 90 pulses). Note: rebound activity following activity suppression by the stimulation.

First, we verified the direct effect of various electric stimulation protocols on the network responses (evoked response; Fig. 5) on day 15 (where all stimulation protocols are delivered for the first time). For the “NoStim” condition, with (L-glutamate) or without (Control) L-glutamate infusion, an overall decrease in network activity was observed (Repeated measure ANOVA, time: p < 0.05; Fig. 5A), which was particularly pronounced 15 min after the start of recording. LFS stimulation increased the network activity of 50% (wMFR in % of baseline firing; 1 min binning; baseline 5 min pre-stimulation), up to the 15-minute mark (Figure A, top panels). Post-LFS, a trend of increased activity was observed in comparison to NoStim control condition (Fig. 5A, top left panel), although these differences were not statistically significant. DCS stimulation showed evoked responses after L-glutamate infusion (Fig. 5A, middle right panel), during Stimulation and Post-stimulation, whereas control condition did not show any effect (Fig. 5A, middle left panel). Combination LFS/DCS stimulation protocols with control and L-glutamate, similarly to LFS, induced a sustained network activity during stimulation and during stimulation/post-stimulation, respectively (Fig. 5A bottom panels). A rhythmic response to LFS was observed in most wells (Fig. 5B; 3 out of 4 wells; 100msec binning for wMRF). Representative peri-stimulus histograms illustrate the transient depression in population firing (wMFR z-score) followed by rebound of network activity stimulated by LFS protocols (Fig. 5C). Altogether, these results confirm a direct evoked response of the neural network to FES and possible short-term impact on neural activity for our protocols.

### Electrical stimulation initiates network recovery following L-glutamate induced inhibition

**Figure 6:**
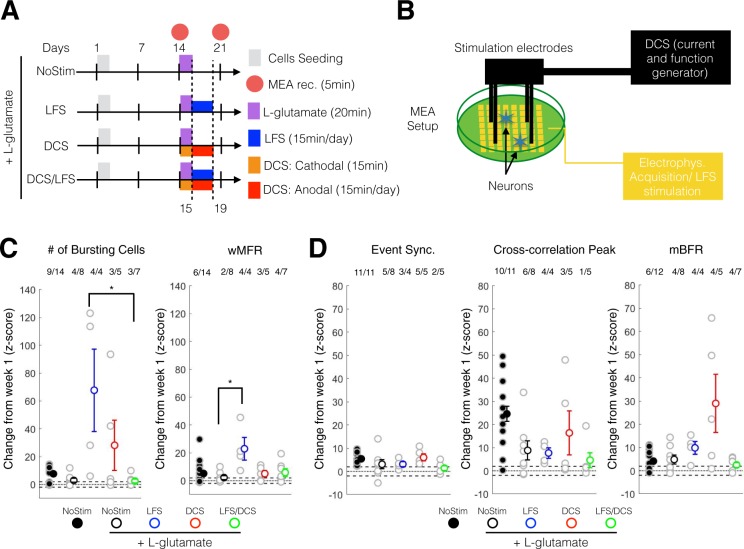
DCS and LFS stimulation enhanced the maturation in excitability and neural synchrony following acute L-glutamate treatment. (**A**) Experimental schedule for ESC-derived neuron seeding, culture and treatment. L-glutamate (100 µM) was administered at day14 for 20 min then washed out. LFS was administered from day 15 to day 19 using 10 µA at 0.1 Hz, 15 min/day. DCS was administered in two phases: day14 a cathodal stimulation was performed for 15 min; from day 15 to day 19, anodal stimulation was administered for 15 min per day. Recording was performed on day 14 (week 2) and day 21 (week 3). (**B**) Schematic set up for electrical stimulation on MEA plate. LFS stimulation (10 µA, 0.1 Hz, 15 min/day) was delivered through the MEA electrode using the Maestro system. Four stainless screws were positioned above the MEA cultured neurons and were used to deliver a controlled current (DCS: single-time 10 µA monophonic cathodal 15 min and daily 10 µA monophonic anodal current, 15 min/day) using a custom battery-powered system. (**C**) Change at week 3 (expressed as a Z-score from week1 baseline) of the number of bursting cells (left panel) and wMFR (right panel). For post-hoc multiple comparison using Dunn-Sidak correction *, ** and *** indicate p < 0.05, p < 0.01 and p < 0.001, respectively. wMFR: weighted mean population firing rate. (**D**) Change at week 3 (expressed as a Z-score from week1 baseline) of the event synchronization (left panel), cross-correlation peak (middle panel) and mBFR (right panel). For post-hoc multiple comparison using Dunn-Sidak correction *, ** and *** indicate p < 0.05, p < 0.01 and p < 0.001, respectively. mBFR: mean population burst firing rate.

We then quantified changes at week-3 following the completion of all protocols (Fig. 6A) relatively to week 1 (Z-score) in order to understand the long-term effect of FES on network maturation.

Following L-glutamate treatment, we found that LFS stimulation significantly increased the rate of maturation in neuronal excitability measures (# of cell bursting; one-way ANOVA, p = 0.039; LFS-LFS/DCS: p = 0.046; post-hoc Dunn correction) and marginally significantly for wMFR (one-way ANOVA, p = 0.065; Sham-LFS: = 0.046; post-hoc Dunn correction) compared to the NoStim L-glutamate group (Fig. 6C). Importantly, following L-glutamate, we found that LFS (Cell bursting and wMFR: 4/4 or 100%) and DCS (Cell bursting and wMFR: 3/5 or 60%) increased network excitability (Cell bursting: 4/8 or 50%; wMFR: 2/8 or 25%) when compared to NoStim. We observed that LFS and DCS after L-glutamate increased the rate of maturation in excitability within or higher than the range of NoStim control, indicating a possible recovery to normal control level.

The DCS group showed an increasing trend with respect to event synchronization (one-way ANOVA, p = 0.364) and cross-correlation peak (one-way ANOVA, p = 0.665) measures of synchrony when compared to the NoStim group (Fig. 6D), although, no significant group differences were observed. The DCS group also showed a marginally significant difference in mBFR when compared to the NoStim group (one-way ANOVA, p = 0.053; Fig. 6D, right panel), with a similar trend to the other measures of synchrony as described above. As for measures of excitability, LFS (Cell bursting: 3/4 or 75%; wMFR: 4/4 or 100%) and DCS (Cell bursting: 5/5 or 100%; wMFR: 3/5 or 60%) groups induced greater cell bursting and wMFR when compared to the NoStim group (Cell bursting: 5/8 or 62.5%; wMFR: 4/8 or 50%). Combination LFS/DCS treatment did not demonstrate any clear effects on these measures.

Altogether, these results indicate that LFS and DCS had a positive impact on the rate of change in excitability and synchrony compared to the NoStim L-glutamate groups, returning within range or higher than NoStim control levels.

### Electrical stimulation following L-glutamate-inhibition significantly enhanced excitability and plasticity related gene expression

In order to understand the underlying effects of electrical stimulation of untreated cells and following L-glutamate treatment, we investigated the change in expression of transcripts encoding N-methyl-D-aspartate (NMDA) receptor subunit A and B (*NR2A* and *NR2B*; transcripts encoding two receptors linked to glutamate excitation and the modulation of long-term potentiation and depression), brain-derived neurotrophic factor (*BDNF*; transcript encoding a protein that promotes synaptogenesis and neurogenesis) and RAS-related protein (*RAB3A*; transcript encoding a protein related to synaptic vesicles transport).

**Figure 7:**
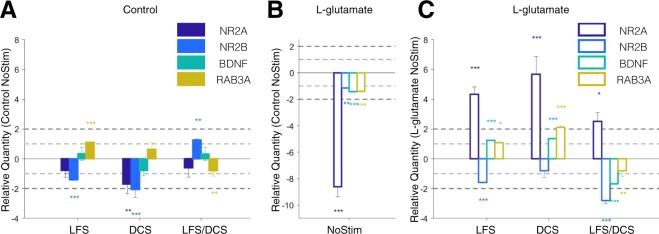
DCS and LFS stimulation alters the expression profile of *NR2A*, *NR2B*, *BDNF* and *RAB3A*. (**A**) Relative quantity (RQ to Control NoStim) of gene expression change in response to LFS, DCS and LFS/DCS stimulation without toxicity at week 3. (**B**) Relative quantity (RQ to Control NoStim) of gene expression change following L-glutamate toxicity at week 3 (L-glutamate NoStim). (**C**) Relative quantity (RQ to L-glutamate NoStim) for gene expression change for LFS, DCS and LFS/DCS stimulation following L-glutamate toxicity at week 3. Data is shown as mean +/−SEM. *, ** and *** indicate p < 0.05, p < 0.01 and p < 0.001, respectively, on two-sample student t-test between test and control samples (n = 6 repeats per group). Light and dark gray dashed lines mark 1- and 2 fold change in expression. Bar plots in order from left to right represent NR2A: NMDA receptor sub unit 2A, NR2B: NMDA receptor sub unit 2B, BDNF: brain-derived neurotrophic factor, RAB3A: RAS-related protein rab3.

The quantitative real-time PCR analysis (fold up and down) showed that under the control condition (no L-glutamate treatment), LFS and DCS stimulation induced a reduction in *NR2A* (LFS: p = 0.270; DCS: p = 0.002) and *NR2B* (LFS: p < 0.001; DCS: p < 0.001) expression when compared to the unstimulated controls (Control NoStim). *RAB3A* expression was significantly increased under LFS (p < 0.001) and decreased for the combination of LFS/DCS stimulation (p = 0.001) when compared to the Control NoStim (Fig. 7A). In contrast to LFS and DCS stimulated groups, the cells exposed to combination LFS/DCS stimulation induced significantly increased expression of *NR2B* encoding transcripts (p = 0.002) when compared to the Control NoStim.

Following L-glutamate treatment, we found that all transcripts encoding excitability and plasticity related proteins were significantly downregulated in the L-glutamate NoStim group when compared to the Control NoStim group (p < 0.01; Fig. 7B), with an ~8 fold downregulation of *NR2A* (RQ: −8.6 ± 0.78;p < 0.001). In contrast, L-glutamate treated cells subjected to electrical stimulation (LFS, DCS, and combination LFS/DCS) significantly upregulated *NR2A* expression (LFS p < 0.001; DCS p < 0.001; LFS/DCS p = 0.012) when compared to L-glutamate NoStim treated controls (Fig. 7C). Interestingly, the gene expression of *NR2B* was significantly decreased in all cells subjected to stimulation protocols (LFS: p < 0.001; DCS: p = 0.457; LFS/DCS: p < 0.001) when compared to L-glutamate NoStim. LFS and DCS also significantly increased the expression of *BDNF* (LFS and DCS: p < 0.001) and *RAB3A* (LFS: p = 0.019; DCS: p < 0.001) following L-glutamate treatment when compared to L-glutamate NoStim, which was in stark contrast to combination LFS/DCS treated group that demonstrated a significant decrease in *BDNF* expression.

In order to better understand the effect of stimulation following L-glutamate treatment, we tested the relative difference in fold change of expression between each stimulation protocol with and without L-glutamate. For all genes except *NR2B*, we detected a significant effect for main factor treatment (two-way ANOVA, treatment: p < 0.05; two levels: control and L-glutamate) and main factor interaction (two-way ANOVA, treatment × stimulation: p < 0.005: stimulation, 3-levels: LFS, DCS and LFS/DCS). *NR2B* only had a significant factor interaction (two-way ANOVA, treatment × stimulation: p < 0.001) and a marginal effect of the main factor treatment (two-way ANOVA, treatment: p = 0.054). These results are consistent with the L-glutamate induced significant downregulation (Fig. 7B), and the subsequent stimulation-induced significant upregulation of these transcripts (Fig. 7C). Post-hoc comparison between control and L-glutamate condition revealed a significant increase for *NR2A* for all stimulation conditions (p < 0.001, Holm-Sidak correction). *NR2B* expression was significantly decreased under L-glutamate treatment compared to control for DCS (P < 0.05, Holm-Sidak correction) and LFS/DCS (P < 0.001, Holm-Sidak correction) groups. *BDNF* expression was significantly increased under L-glutamate treatment compared to control for DCS and LFS/DCS (P < 0.001, Holm-Sidak correction) groups. Under L-glutamate treatment, only the DCS condition evoked the increased expression of *RAB3A* when compared to control.

Notably, following L-glutamate (RQ to L-glutamate NoStim), post-hoc multiple comparison indicated that LFS and DCS induced significantly higher expression of *NR2A* (p < 0.05, Holm-Sidak correction), *BDNF* (p < 0.001, Holm-Sidak correction) and *RAB3A* (p < 0.001, Holm-Sidak correction) than the combination LFS/DCS group. The expression of *NR2B*, however, was significantly decreased by the combination LFS/DCS (p < 0.05, Holm-Sidak correction) following L-glutamate exposure.

Altogether these results indicate that the DCS and LFS alone following L-glutamate treatment induced the significant up-regulation of excitability (*NR2A*) and plasticity (*BDNF* and *RAB3A*) related genes, whereas LFS/DCS combination had adverse effects.

## Discussion

In our current work, we present a first evaluation of direct current stimulation protocols on the maturation of neuronal networks following acute glutamate exposure. We demonstrated using ESC-derived neuron and glia co-cultures on MEAs, that the glutamate-induced impairment in maturation (Fig. 4) can be overcome using direct current stimulation (DCS and LFS; Fig. 6) through potential mechanisms that involve the change in expression of excitability and plasticity-related genes *NR2A/B*, *BDNF* and *RAB3A* (Fig. 7). These studies provide: 1) A proof-of-concept of the utility of MEAs as a platform to investigate the effects of FES following excitotoxicity in physiologically relevant conditions; and 2) validation that DCS and LFS can be used to evoke network maturation and recovery following acute glutamate treatment.

### Model for glutamate-induced excitotoxicity in ESC-derived co-cultures

Glutamate is one of the most common excitotoxic agents responsible for the propagation of secondary neuronal damage after TBI^75^. Acute glutamate release (>300 µMol) reportedly results in cell swelling and neurotoxicity due to NMDA-receptor binding^76,77^ and the failure of glutamate re-uptake by astrocytes^78,79^ in cortical neuron and glial co-cultures. Previous MEA-based studies showed a dose-dependency to L-glutamate in network response and mostly focused on a single time point change in network excitability or connectivity^44,71,80–82^. Our results corroborate these previous findings, and further demonstrate that acute glutamate treatment (single dose 100 µM of L-glutamate for 20 min) resulted in reduced network rate of maturation in excitability (Fig. 4C) and connectivity (Fig. 4D) in ESC-derived neuron and glial co-cultures, similarly to embryonic cortical neuron and glial co-cultures, without inducing any significant difference in the change in number of active electrodes at week 3 (i.e. lesser impact on the viability of the neural cells; repeated-measure ANOVA, group × time: p = 0.298) or silencing^71^. Acute exposure to L-glutamate might cause delayed and hour-long increase in intracellular calcium concentration^83^ and in turn might modulate plasticity changes^84,85^. In this study, we have successfully demonstrated immediate (within minutes) and extended (1 week) changes in network rate of maturation following acute glutamate exposure (Fig. 4) and its corresponding long-term modulation on plasticity-related gene expression (Fig. 7B), which to our knowledge, has not been reported previously. These results validate the application of MEA recording/stimulation platforms for the high-throughput and longitudinal assessment of cellular and neuronal network maturation, and correlative gene expression in response to excitotoxic stimuli *in vitro*.^86,87^

### Acute L-glutamate and NR2A/B expression

NMDA subunit composition and postsynaptic location of NMDARs are critical determinants of synaptic plasticity^88^. NR2A receptors are synaptically located and associated with long-term potentiation (LTP), while NR2B receptors are extrasynaptically located and associated with long-term depression (LTD). Most importantly, the ratio of NR2A/NR2B is thought to be predictive of plasticity changes as observed in brain development studies^89–91^. We observed that in our control condition, the general expression level of *NR2A* and *NR2B* transcripts decreased following LFS and DCS, whereas combination LFS/DCS increased *NR2B* expression. Importantly, following acute L-glutamate treatment, the L-glutamate treated NoStim group significantly down-regulated all transcripts, and particularly* NR2A* in comparison to the control NoStim condition (Fig. 7B), which might be consistent with NMDA receptor-mediated calcium influx and neuronal over-excitation^92^. Interestingly, we found a significant up-regulating (*NR2A*, *BDNF* and *RABA*) and down-regulating (*NR2B*) effect of stimulation when coupled with L-glutamate treatment (Fig. 7; two-way ANOVA, treatment factor: p < 0.001; interaction treatment × stimulation: p < 0.004). Indeed, both LFS and DCS resulted in a significant up-regulation of *NR2A* (p < 0.001) expression and down-regulation of *NR2B* (LFS: p < 0.001) compared to L-glutamate NoStim condition, which was consistent with the observed improvement in the network maturation over one week period (week3). These results support the use of LFS and DCS post injury-induced neurotoxicity for the recovery of neuronal network^12,74,93^, but also provide evidence that the preventive use of these stimulation regimens could protect from neurotoxicity by down-regulating NMDA-receptor expression^92,94,95^.

### Evoked response to FES and translational implications

In control conditions, the LFS protocol evoked the significantly increased activity of maturing neural networks during stimulation, whereas DCS and LFS/DCS combination had a milder impact. This difference could arise from the nature of our setup where LFS stimulation was performed directly through proximal electrodes (10 uA; Iridium electrodes, 120 um diameter), while DCS stimulation was performed through more distant electrodes submerged in the media (10 uA; stainless steal electrodes, 1.5 mm diameter). We did not perform all stimulations through the MEA electrodes in order to allow for simultaneous recording and stimulation, and obtain the quantification of neuronal evoked response. Despite differences in the evoked responses, DCS and LFS/DCS evoked sustained neural activity during stimulation (10 min mark after stimulation start) and post-stimulation (Fig. 5A, right panels), and preferentially after L-glutamate treatment. These results suggest that DCS-based stimulations at low current could provide enhancement of maturation in neural network connectivity (Fig. 6D) without evoking significant changes in neural activity during stimulation, which is most desired in clinical settings. Notably, LFS resulted in network entrainment (Fig. 5B; 0.1 Hz) that stopped with stimulation and that was not evident in network rhythmic synchronization.

### DCS and LFS likely improved network maturation following glutamate-induced impairment by upregulating plasticity genes

Cathodal/anodal tDCS and LFS are two of the most commonly applied non-invasive stimulation approaches reported in the pre-clinical and clinical literature^1^ for the treatment of brain injury and neurodegenerative diseases. These two protocols have not been extensively compared and their mechanisms of action for treating brain disorders/injury are poorly understood. We found that LFS significantly increased the rate of maturation in excitability (# of cell bursting: p < 0.05, and wMFR: p = 0.065; 100% of well showing increase versus <50% for the sham group) and also promoted a marginal increase in synchrony maturation as seen through cross-correlation peak and mBFR (mean bursting firing rate; p = 0.05; 100% of well significantly increasing versus <75% for the sham group). Since LFS has been shown to favor long-term potentiation (LTP) through the Trek/BDNF pathway^16^, we investigated its effects on other related network plasticity-inducing genes. At the electrophysiological level, we found that LFS could help sustain network activity (Fig. 5A) during stimulation,and the effect of which did not persist post-stimulation (Fig. 5A, Post-stimulation period). Interestingly, LFS alone in the control condition promoted *RAB3A* expression without inducing a corresponding increase in *BDNF* expression (Fig. 7A). However, following L-glutamate treatment, LFS significantly up-regulated both *BDNF* and *RAB3A* (Fig. 7B), which also corresponded to an improved network connectivity as seen through our synchrony measures (Fig. 6D). *RAB3A* is an intracellular vesicular trafficking protein required for calcium exocytosis and is thought to be required (i.e. up-stream) for the BDNF-dependent increase in synaptic plasticity^96^. Therefore, RAB3A-BDNF might corroborate the effect of LFS on the plastic change observed following L-glutamate excitotoxicity.

Similarly to LFS, we observed that DCS following L-glutamate treatment showed a trend of increased excitability (Fig. 4C; # of bursting cells) and a clear, although non-significant, increase in rate of maturation in synchrony (Fig. 4D; Cross-correlation peak and mBFR) as well as increased *BDNF* and *RAB3A* expression. Surprisingly, DCS in control condition induced a significantly reduced expression of *NR2A* and *NR2B* expression without a specific effect on *BDNF* or *RAB3A* (Fig. 7A), but without inducing significant effects on network maturation (one-way ANOVA, p > 0.05 for all measures; Supplementary Fig. 4B,C). This result does not corroborate our observation that DCS might induce long-lasting activation of neural network (Fig. 5A) and previous *in vivo* studies that suggest a possible increase in plasticity and memory through an epigenetic modulation of BDNF expression by tDCS^65,97^. However, it is important to note that the DCS stimulation used in this study was a combination of cathodal and chronic anodal stimulations and was tailored for use following glutamate treatment as opposed to one-time anodal excitatory stimulation^65^. Overall, our results confirm that the therapeutic use of LFS and DCS mechanistically contributes to the enhancement of network maturation consistently with an up-regulation of plasticity gene expression.

### LFS/DCS demonstrated negative effects on molecular and network plasticity following L-glutamate treatment

Since DCS and LFS are two promising neurostimulation methods for the modulation of learning and memory^98^, we sought to test the combination of the two protocols on MEA neural cell co-cultures. Surprisingly, in control condition combining DCS and LFS resulted in down-regulation of *BDNF* and an up-regulation of *NR2B* expression, which was supported by no significant change in network synchrony or excitability (one-way ANOVA, p > 0.05; Supplementary Fig. 4C). Following acute L-glutamate, LFS/DCS increased *NR2A* and down-regulated *NR2B* expression, in addition to strongly suppressing *BDNF* and *RAB3A* expression. Consistent with our qRT-PCR results, the administration of LFS/DCS following L-glutamate treatment, LFS/DCS combination did not induce changes in the rate of maturation and excitability (Fig. 6D), although evoked responses from the network were observed during and post-stimulation (Fig. 5A). These results suggest that LFS and DCS when used in combination (LFS/DCS) may have induced neuronal over-excitation (Fig. 5A) and fatigue leading to the negative results observed. Future studies shall include a better correspondence and comparison of electrical stimulation methods^46^, which would in turn likely provide optimal stimulation parameters for *in vivo* applications.

## Electronic supplementary material

Supplementary Figures
