# Supplementary material for: Chronic Electrical Stimulation Promotes the Excitability and Plasticity of ESC-derived Neurons following Glutamate-induced Inhibition In vitro
Source: Sci Rep. 2018 Jul 19;8:10957. doi: 10.1038/s41598-018-29069-3 (PMC6053382; doi:10.1038/s41598-018-29069-3)
Supplement: Supplementary file 1 — Supplementary Figures [file 41598_2018_29069_MOESM1_ESM.pdf]

Chronic Electrical Stimulation Promotes the Excitability and Plasticity of ESC-derived Neurons following Glutamate-induced Inhibition *In vitro*

Charles-Francois V. Latchoumane[1], LaDonya Jackson[1], Mohammad .S Eslampanah Sendi[3], Kayvan F. Tehrani[1,2], Luke J. Mortensen[1,2], Steven L. Stice[1], Maysam Ghovanloo[3], Lohitash Karumbaiah[1*]

[1] Regenerative Bioscience Center, ADS Complex, University of Georgia, Athens, Georgia

[2] School of Chemical, Materials, and Biomedical Engineering, College of Engineering, University of Georgia, Athens, Georgia

[3]School of Electrical and Computer Engineering, Georgia Institute of Technology, Atlanta, Georgia

***Corresponding Author:**
Lohitash Karumbaiah, Ph.D.
Assistant Professor of Regenerative Medicine
Regenerative Bioscience Center
The University of Georgia
ADS Complex, 425 River Road
Athens, GA 30602-2771
Phone: 706-542-2017
Fax: 706-542-2017
Email: lohitash@uga.edu

Supplementary Figures:

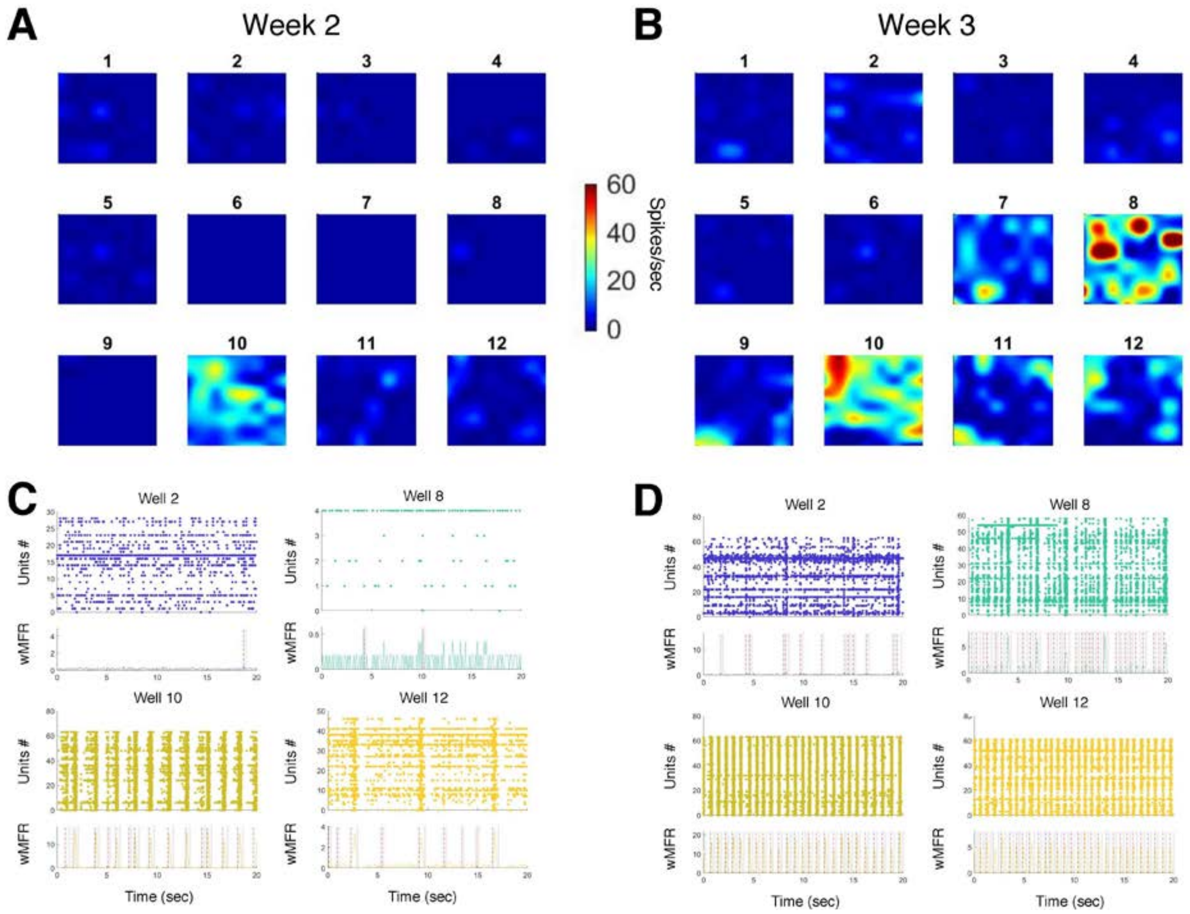

Supplementary Figure 1: Representative network activity in control condition

(**A**) Average activity map (spikes/sec) for 12 wells recorded under control conditions on week 2. Average activity is estimated for each electrode separately over a period of 5min. (**B**) Average activity map (spikes/sec) for 12 wells recorded under control conditions on week 3. Average activity is estimated for each electrode separately over a period of 5min. (**C**) Raster plot of 4 wells activity recorded at week 2 (top panel) and the wMFR (bottom panel; spikes/bin/unit; bin size: 100 msec); 20-sec epoch. The vertical dashed and dotted lines indicate the start and stop of a detected population burst, respectively. (**D**) Raster plot of 4 wells activity recorded at week 3 (top panel) and the wMFR (bottom panel; spikes/bin/unit; bin size: 100 msec); 20-sec epoch. The vertical dashed and dotted lines indicate the start and stop of a detected population burst, respectively.

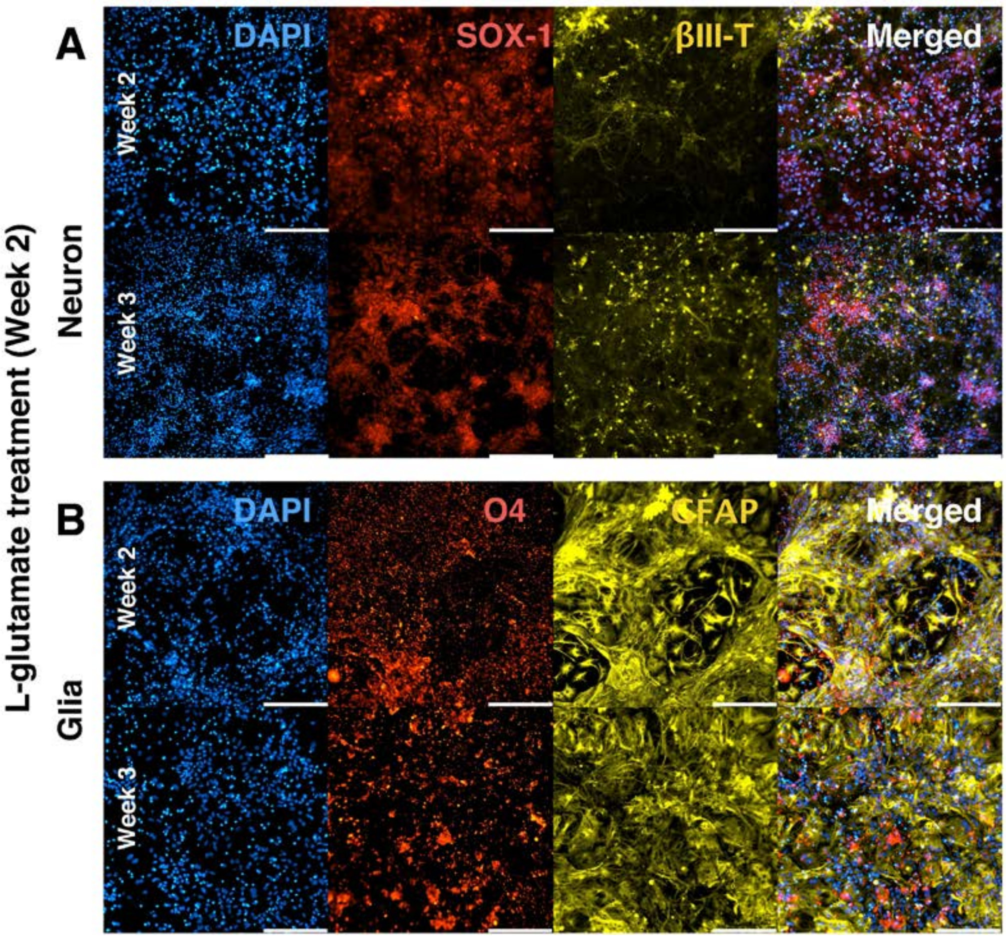

Supplementary Figure 2: ESC-derived neurons and glia matured into neural tissue after L-glutamate cytotoxicity (week2)

(**A**) ESC-derived neurons population for the L-glutamate treated group at week 2 (top panels; immediately after cytotoxicity delivery) and week 3 (bottom panels). From left to right panels are shown DAPI, SOX-1 (Neural progenitor origin marker), βIII-Tubulin (Neuronal marker) and merged image for all 3 fluorescent markers. The scale bar is for 200 µm. (**B**) ESC-derived glial population for the L-glutamate treated group at week 2 (top panels; immediately after cytotoxicity delivery) and week 3(bottom panels). From left to right panels are shown DAPI, O4 (Oligodendrocyte marker), GFAP (Glial marker) and merged image for all 3 fluorescent markers. The scale bar is for 200 µm.

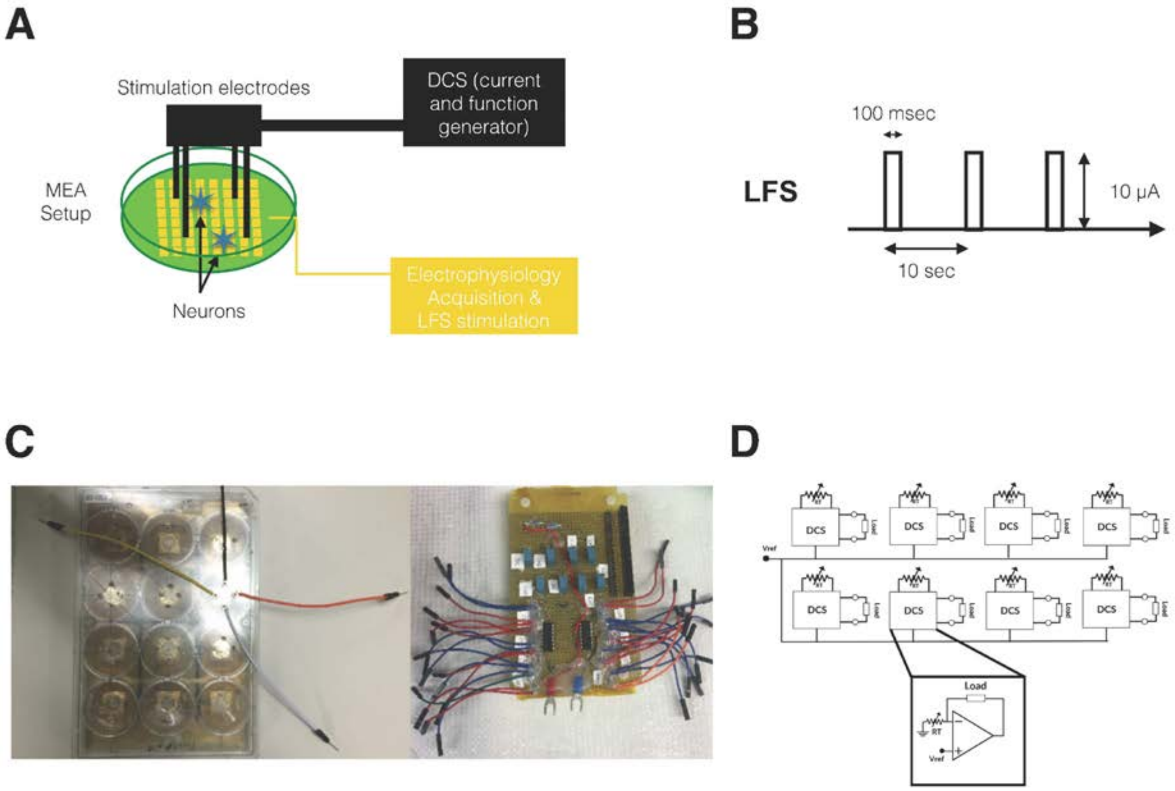

Supplementary Figure 3: *In vitro* stimulation set up
(**A**) Schematic setup for electrical stimulation on MEA plate. LFS stimulation (10 µA, 0.1 Hz, 15 min/day) where delivered through the MEA electrode using the Maestro systems. Four stainless steel screws were positioned above the MEA cultured neurons and delivered a controlled current (DCS: 10 µA monophasic cathodal 15 min and 10 uA monophasic anodal current, 15 min/day) using a custom battery-powered system. (**B**) Parameters used for the LFS protocol. LFS was delivered at 0.1 Hz at an intensity of 10uA for 15 min every day for 5 days. The pulse width was set to 100 msec. (**C**) Custom-made DCS delivery system. The MEA cover plate was customized to host 4 stainless screws for DCS delivery (left panel) with a current distribution controlled through a microcontroller (right panel). (**D**) Block diagram of the custom eight-channel direct current stimulation (DCS) system. The design supports the control of current passing through the load (i.e. a mixed population of neurons and glia) using a variable resistor (RT). The schematic of DCS block is shown in inset which utilizes a negative feedback on the inverting input of the op-amp (MC33204P). As a result, the amount of current passing through the load is proportional to V_{ref}. Using this configuration, anodal and cathodal currents were set by changing the polarity of load.

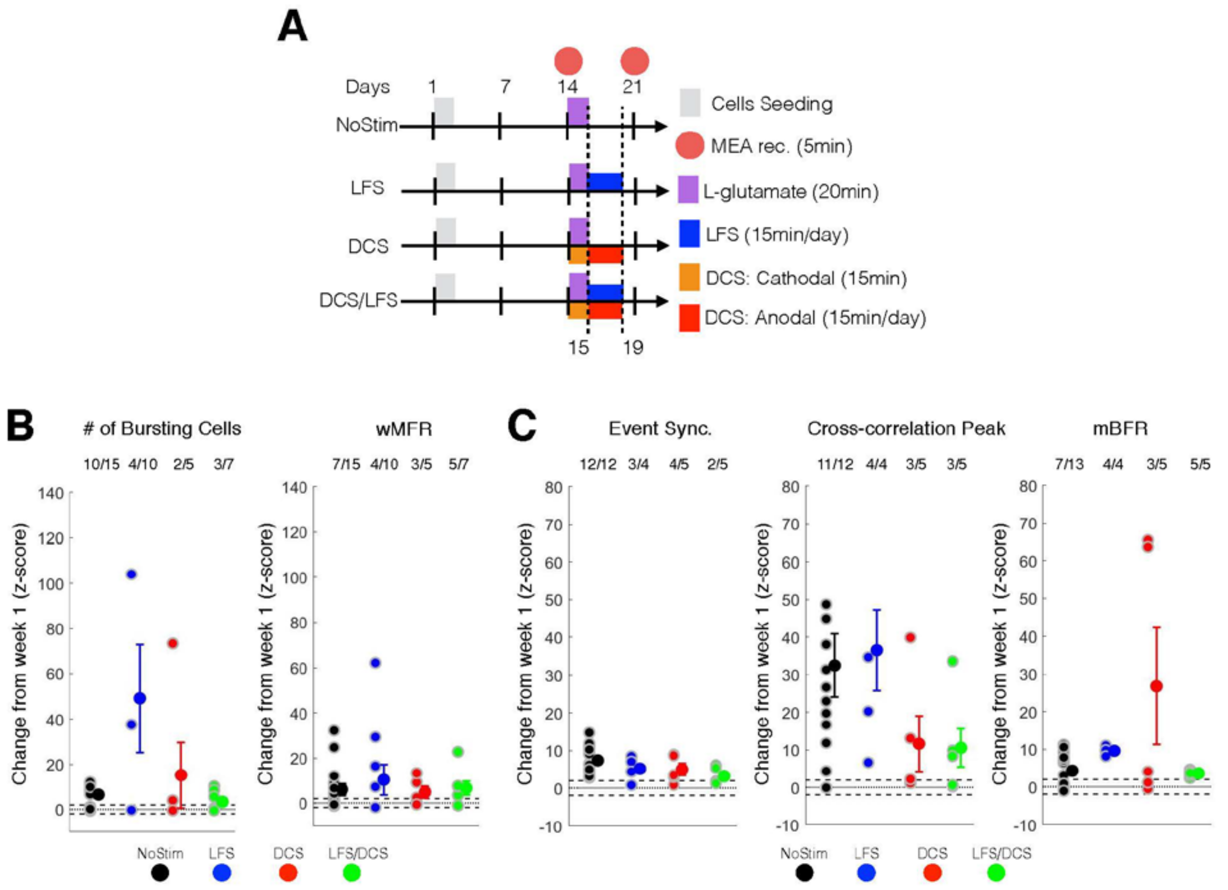

Supplementary Figure 4: DCS/LFS stimulation did not affect maturation in control condition

(**A**) Experimental schedule for ESC-derived neuron seeding, culture and treatment. (**B**) Change at week 3 (expressed as a Z-score from week1 baseline) of the number of bursting cells (left panel) and wMFR (right panel) for the control condition. For post-hoc multiple comparison using Dunn-Sidak correction *, ** and *** indicate $p<0.05$, $p < 0.01$ and $p <0.001$, respectively. wMFR: weighted mean population firing rate. (**C**) Change at week 3 (expressed as a Z-score from week1 baseline) of the event synchronization (left panel), cross-correlation peak (middle panel) and mBFR (right panel) for the control condition. For post-hoc multiple comparison using Dunn-Sidak correction *, ** and *** indicate $p<0.05$, $p < 0.01$ and $p <0.001$, respectively. mBFR: mean population burst firing rate.
